# Post-Arthroplasty Spatiotemporal Gait Parameters in Patients with Hip Osteoarthritis or Developmental Dysplasia of the Hip: An Observational Study

**DOI:** 10.3390/jfmk9030110

**Published:** 2024-06-25

**Authors:** Sophia Stasi, Georgios Papagiannis, Athanasios Triantafyllou, Panayiotis Papagelopoulos, Panagiotis Koulouvaris

**Affiliations:** 1 1st Department of Orthopaedic Surgery, National and Kapodistrian University of Athens, 12462 Athens, Greece; athanat@gmail.com; 2 Biomechanics Laboratory, Department of Physiotherapy, University of the Peloponnese, 23100 Sparta, Greece; grpapagiannis@yahoo.gr (G.P.); pjportho@med.uoa.gr (P.P.); info@drkoulouvaris.gr (P.K.)

**Keywords:** orthopedics biomechanics, spatiotemporal gait parameters, functionality, total hip arthroplasty, hip osteoarthritis, developmental hip dysplasia

## Abstract

Total hip arthroplasty (THA) is a preferred treatment for primary osteoarthritis (OA) or secondary degenerative arthropathy due to developmental hip dysplasia (DDH). Gait analysis is considered a gold standard for evaluating post-arthroplasty walking patterns. This study compared post-THA spatiotemporal gait parameters (SGPs) between OA and DDH patients and explored correlations with demographic and clinical variables. Thirty patients (15 per group) were recorded during gait and their SGPs were analyzed. Functionality was evaluated with the Oxford Hip Score (OHS). The OA patients were significantly older than DDH patients (*p* < 0.005). Significant and moderate to strong were the correlations between SGPs, age, and four items of the OHS concerning hip pain and activities of daily life (0.31 < Pearson’s r < 0.51 all *p* < 0.05). Following THA, both groups exhibited similar levels of the examined gait parameters. Post-arthroplasty SGPs and OHS correlations indicate limitations in certain activities. Given the absence of pre-operative data and the correlation between age and SGPs and OHS, ANCOVA testing revealed that age adjusts OHS and SGP values, while pre-operative diagnosis has no main effect. These findings indicate that hip OA or DDH do not affect postoperative SGPs and patients’ functionality. Future studies should examine both kinematic and kinetic data to better evaluate the post-THA gait patterns of OA and DDH patients.

## 1. Introduction

Total hip arthroplasty (THA) is the treatment of choice for end-stage arthritic hip conditions that cause chronic pain, discomfort, and significant functional impairment [1]. Among the pathological conditions that lead to THA are primary osteoarthritis and secondary degenerative arthropathy due to developmental dysplasia of the hip (DDH) [1].

Osteoarthritis (OA) is the most common type of primary degenerative arthropathy and is a major cause of chronic disability [2]. OA is the clinical and pathological outcome of a sequence of biological and metabolic processes of joint components/tissues, and is associated with structural alterations, such as degeneration of articular cartilage and changes in the subchondral bone. It ultimately leads to the limitation or abolition of the functionality joint as a kinetic and—in the case of the hip joint—supporting skeleton unit [3]. On the other hand, in DDH, the acetabulum and femur are underdeveloped, the femur adapts to an abnormal position, and the soft tissues of the area are shortened [4]. Leg-length discrepancy (LLD), decreased hip abduction range of motion, positive Trendelenburg sign, and shortened iliopsoas and hip adductor muscles are often seen in patients with DDH, while usually they walk with plantar flexion (toe support) [4]. These patients develop symptomatic secondary OA in their fourth or fifth decade of life, and a large number of them are forced to resort to THA at a younger age than patients with primary OA [5,6].

Gait assessment is an essential measure of postoperative outcomes after THA, as gait is a crucial indicator of the level of functional recovery [7,8]. Gait analysis is used to assess gait patterns in different groups of patients [9]. Specifically, spatiotemporal gait parameters are a way to objectively assess dysfunctional gait and monitor treatment progress in a clinical setting [10]. They are also considered a valuable adjunct to clinical and radiological assessment [9].

In the gait analysis of hip OA patients, several non-physiological features can be observed that result from the main symptoms of osteoarthritis. The most characteristic change is reduced gait speed with the gait pattern, including LLD [11]. A systematic meta-analysis, which included 30 studies that studied spatiotemporal characteristics in hip OA patients, reported that the selected walking speed and the average walking pace were slower compared to healthy individuals. At the same time, the step and stride lengths were shorter, the double support phase was shorter, and the step width was larger than those of their healthy peers [10]. Similarly, in gait analysis studies where the gait pattern of DDH patients has been studied, it has been reported that in relation to healthy peers, they walk with a reduced gait speed [12,13,14], have a shorter step length [12,14], and the affected limb shows a longer double support time and a shorter single leg support time [12,13]. Postoperatively, gait pattern improves significantly in all patients regardless of preoperative diagnosis [15]. Nevertheless, even ten years after undergoing THA surgery, it has been reported that patients’ walking ability does not reach the same levels as their peers of the same age [16].

However, the literature review revealed that in most gait analysis studies concerning the post-THA gait, either in patients with hip OA or DDH patients, the comparison was carried out with non-operated [17,18] or with healthy peers [16,19]. Up to our knowledge, only one study compares post-arthroplasty gait parameters between patients with primary OA and DDH patients [20]. Therefore, the present study aims to compare the post-arthroplasty gait spatiotemporal parameters in patients with primary OA and patients with DDH. The primary study hypothesis is that the distinct pathomechanics associated with each condition contribute to the preoperative adaptations in gait. Consequently, it is thought that there may be variations in the improvement of postoperative spatiotemporal characteristics. Secondary outcomes included potential correlations between postoperative spatiotemporal and demographic/clinical characteristics relating to patients’ functionality. Based on previous relevant studies on post-THA patients [21,22], we hypothesized that postoperative spatiotemporal characteristics have the same trend with patient-reported outcomes. Biomechanists and rehabilitation experts could utilize such evidence to advance the development of targeted rehabilitation programs that ultimately improve the functional capacity of patients.

## 2. Materials and Methods

### 2.1. Trial Design

This research was conducted in a biomechanics laboratory (Ethics Approval No: 42609/05-05-2022). Patients who agreed to participate in the study were given written informed consent according to the principles of the Declaration of Helsinki and its later amendments [23]. The present study conformed to the “Strengthening the Reporting of Observational Studies in Epidemiology” (STROBE) statement for reporting observational studies [24] (Supplementary File).

### 2.2. Participants

Patients over 45 were included in the present study, as it has been reported that one in four arthroplasties performed before age fifty are due to hip dysplasia [25]. The patients were required to have undergone primary THA three to five years prior to their enrollment in the present study; namely, the THA surgery must have been performed from January 2019 to December 2021. This postoperative time was chosen as a sufficient period to allow all patients to adopt a stable gait pattern [26]. All participants underwent a cementless THA through a posterior approach [27,28] performed by the same team of orthopaedic surgeons and all patients followed the same postoperative physiotherapy program. Information was obtained by reviewing the registry data from their admission for THA surgery and by conducting telephone interviews. After the first screening, the enrolled patients were divided into two groups according to their preoperative diagnosis. The first group (OA group) included patients who underwent THA due to unilateral hip OA and the second group (DDH group) included patients who underwent THA due to secondary degenerative arthropathy due to unilateral DDH. Patients were excluded from the study if they had had previous hip joint-preserving procedures or acquired post-THA a leg-length discrepancy (LLD) greater than 2 cm, a nerve injury, a history of other orthopedic surgery on the lower limbs or spine, declared that they suffered from a severe balance disorder, any neurological and musculoskeletal diseases that prevented them from performing free walking, or used a walking aid.

### 2.3. Outcomes

Initially, the demographic characteristics (age, gender, height, weight, and body mass index) of the two groups’ populations were recorded. The preoperative grade of hip OA was recorded according to the Kellgren–Lawrence classification system [29] and the grade of DDH according to the Crowe classification system [30]. Anthropometric data were collected using a Seca scale (model 803) and a height meter. The knee and ankle joints’ diameters, anterior superior iliac spine (ASIS) distance, and pelvic depth were measured with a caliper.

Patients’ functionality was measured using the Oxford Hip Score (OHS), which consisted of 12 questions assessing pain and function during activities of daily living (ADLs). The OHS questionnaire was designed and developed to assess patients undergoing THA [31]. Items’ response scores range from 0 points (most severe symptoms) to 4 points (least symptoms), with a total score between 40 and 48 indicating satisfactory joint function [32].

### 2.4. Instrumentation and Procedure

A motion recording system with six Vicon MCam optoelectronic cameras (Oxford MetricsGroup Ltd., Oxford, UK) was used to record the patients’ spatiotemporal parameters, which were recorded during walking. 

The equipment was calibrated every morning by the same biomechanist before the measurements, according to the applicable local protocols, to ensure accuracy and enable the calculation of each marker’s three-dimensional (3D) coordinates. The mean error in calculating the difference between the measured and actual distance of two markers fixed to the ends of a rigid rod 600 mm apart was within 0.3 mm. The calibrated volume for this application was 10 m in length (x-axis of the laboratory reference system), 3 m in height (y-axis of the laboratory reference system), and 3 m along the z-axis of the laboratory reference system. Records of these checks and associated calibrations were saved along with all session data. 

**Figure 1:**
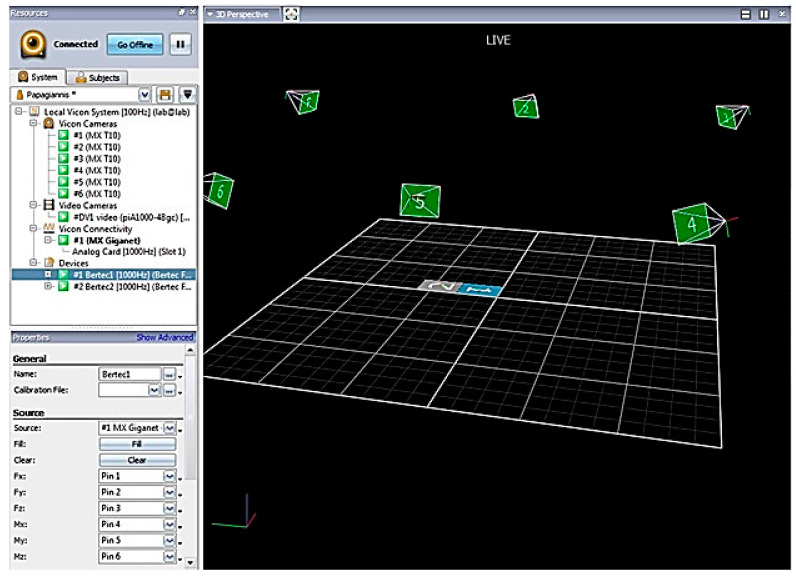
The ViconNexus software version 2.3 figure of data acquisition procedure, depicting laboratory dimensions, six optoelectronic cameras, and two force plates).

All six optoelectronic cameras also used a frequency of 120 Hz for data acquisition, while the motion analysis system error was <0.1 mm in a 10 × 3 × 3 m laboratory space volume (Figure 1). These calibration parameters also ensured the accuracy of the recorded data.

### 2.5. Modeling—Placement of Markers

Motion modeling is an essential concept in the field of biomechanical data recording. The Plug-in Gait marker fitting procedure was employed due to this rationale [33]. Markers were strategically positioned in the anatomical areas of the pelvis and lower extremities. The pelvic markers were placed at the anatomical landmarks of the left anterior superior iliac spine (LASI marker), the right anterior superior iliac spine (RASI marker), the left posterior superior iliac spine (LPSI marker), and the right posterior superior iliac spine (RPSI marker). As for the lower extremities, both the left and right, the following markers were positioned: on the upper lateral 1/3 area of the left and right thigh (LTHI/RTHI markers), on the flexion-extension axis of the left and right knee (LKNE/RKNE markers), and on the lower 1/3 area of the left/right shank (LTIB/RTIB markers). To reconstruct the foot section, markers were positioned on the left/right lateral malleolus, passing along an imaginary line across the left/right transmalleolar axis (LANK/RANK markers), on the left/right calcaneus bone (LHEE/RHEE markers), and on the left/right second metatarsal head, on the mid-foot side of the equinus break between the fore-foot and mid-foot (LTOE/RTOE markers).

To achieve precise localization and positioning of knee markers (LKNE and RKNE), a slight passive flexion and extension of the knee were performed while carefully observing the lateral knee joint skin area. The location where the knee joint’s axis intersects the knee’s outer surface was identified by locating the layer of skin on the thigh that moved the least. This landmark was designated with a pen as the focal point for the rotational movement of the foot’s bottom. 

Thigh markers (LTHI and RTHI) are utilized to identify the location of the knee flexion axis. The LTHI marker was positioned on the lower one-third of the outside lateral area of the thigh, while the RTHI marker was put on the upper one-third of the outer lateral surface of the thigh, slightly under the arm’s reach point. However, the exact height of the markers is not an essential factor in this measurement. Proper identification of the knee flexion axis relies on the reflectors’ anteroposterior location. The thigh marker’s location was modified to align with the plane, including the hip and knee joints center and the axis representing knee flexion and extension.

The alignment of the plantar flexion axis is determined using tibial markers, namely the LTIB and RTIB. The LTIB marker was positioned on the lower one-third of the tibial surface, while the RTIB marker was placed on the upper one-third of the tibial surface, like the thigh markers. The tibial marker was positioned inside the plane, including the center of the knee and ankle joints and the axis representing ankle flexion and extension.

The participants conducted the walking process during a single laboratory session. They were instructed to walk in a manner that closely resembled their usual walking style, with occasional cues given, for a distance of approximately 6 m at a self-chosen tempo. A preliminary static trial was conducted to establish the orientations of the markers before processing the model. Subsequently, participants performed two dynamic trials to familiarize themselves with the testing processes. Ultimately, they completed three additional trials that were considered sufficient and were then analyzed to obtain the representative values of the spatiotemporal parameters [19].

### 2.6. Data Synthesis

Anthropometric measurements were combined with data from markers’ deflections. All markers’ location data were captured using Nexus 2.3 software. The spatiotemporal parameters measured in this study were walking speed, cadence, double support time, single support, step time and length, and stride time and length.

The above spatiotemporal parameters were included in the statistical analysis and were calculated using inverse dynamics and normalization in terms of body mass and length [34].

### 2.7. Statistical Analysis

Data were expressed for continuous variables as mean ± standard deviation (SD) and for categorical variables as frequencies (percentages).

Normality was assessed by Q-Q plot inspection. Pearson’s r correlation index assessed correlations between continuous variables (demographic, clinical, and spatiotemporal parameters’ data) of all patients. Group differences assessed using ANVOVA.

All tests were two-sided, with the significance level being *p* = 0.05. All tests were performed using SPSS v.29 (IBM Corporation, Somers, NY, USA).

## 3. Results

### 3.1. Participants

**Figure 2:**
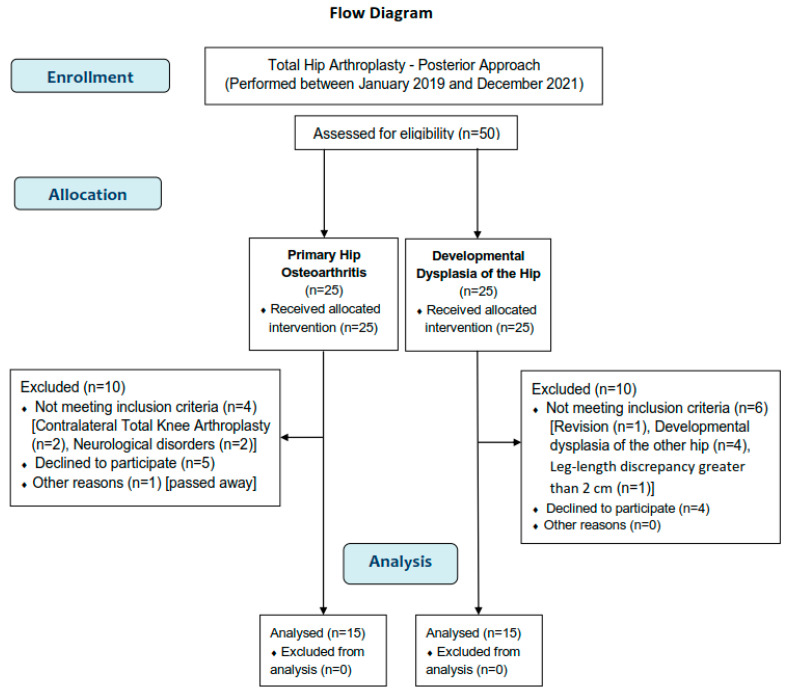
This study’s flow diagram.

A total of 50 patients were enrolled in the present study (the minimum required total sample after Power Analysis was found to be 29 subjects). Of the 50 patients, 25 were diagnosed with hip OA before THA and 25 had unilateral DDH. Ten did not meet the inclusion criteria, nine refused to participate, and one hip OA patient had passed away because of a cause unrelated to THA. Finally, 30 patients (15 in each group) were included. The detailed procedure of the participants’ selection is presented in a flow diagram (Figure 2).

### 3.2. Demographic and Clinical Characteristics

The Q-Q plot inspection revealed that variables had normal distribution; hence, parametric testing was performed. The mean ± SD of the demographic and clinical characteristics of the study’s sample are presented in Table 1. There were no significant demographic or clinical differences between the groups, except for age (*p* < 0.005). When the THA was performed, the mean age of the OA group was 60.1 years (min = 53, max = 68), and the mean age of the DDH group was 46.13 years (min = 36, max = 55 years). The OA group included five men and ten women, while the DDH group consisted of three men and 12 women. Eight patients of the OA group underwent THA due to grade III and seven due to grade IV hip OA, according to the Kellgren–Lawrence classification system. According to the Crowe classification system, the DDH group included four patients with Crowe II, six with Crowe III, and four with Crowe IV dysplastic hip. The greater preoperative LLD of the DDH group was 5 cm, while the hip OA preoperative LLD was not reported in the record files. The means of the post-THA time period were for the hip OA group 3.91 years (min = 3.3, max = 5) and for the DDH group 3.69 years (min = 3.1, max = 4.8).

### 3.3. Correlation Analysis

The correlation analysis revealed significant moderate to strong correlations. Specifically, moderate and positive were the correlations between age and walking speed, step length, and total OHS score (r = 0.31, *p* = 0.00, r = 0.34, *p* = 0.00, and r = 0.36, *p* = 0.04. respectively), while negative and strong was the correlation between age and step time (r = −0.51, *p* = 0.04). Positive and moderate correlations were found between walking speed and item 5 of the OHS (“Could you do the household shopping on your own?”) (r = 0.40, *p* = 0.02) and between cadence and item 11 of the OHS (“How much has pain from your hip interfered with your usual work, including housework?”) (r = 0.38, *p* = 0.03). The single support time positively and moderately correlated with item 5 of the OHS (r = 0.46, *p* = 0.01). Strong and negative was the correlation between step time and item 5 of the OHS (r = −0.51, *p* = 0.00). The step length was moderately and negatively correlated with item 4 of the OHS (“Have you been able to put on a pair of socks, stockings or tights?”) (r = −0.41, *p* = 0.02), while the stride length was moderately and negatively correlated with item 6 (“For how long have you been able to walk before the pain in your hip becomes severe (with or without a walking aid)?”) (r = −0.36, *p* = 0.04) and with item 4 (r = −0.44, *p* = 0.01) of the OHS.

### 3.4. Group Differences

Given the absence of pre-THA data, the correlations between age and SGPS and OHS group differences were assessed using analysis of covariance (ANCOVA).

After controlling for age revealed (Pillai’s trace = 0.05, Wilk’s Lamba = 0.05) the ANCOVA showed that age adjusts the values of the outcomes (SPGs and OHS). Additionally, the multivariate test for the OA and DDH groups (Pillai’s trace = 0.651, Wilk’s Lamba = 0.651) indicated no significant main effect amongst the independent groups of the outcome mentioned above when controlling for age. The above indicates that age does indeed adjust the results, but there are no statistically significant main effects between the two groups, which is a significant finding.

### 3.5. Outcomes

The OHS total score ranged from 38 to 42 in the OA group and 37 to 42 in the DDH group. The mean ± SD of the item scores and the total Oxford Hip Score of both groups are included in Table 2. No significant statistical differences were also observed between the two groups regarding their spatiotemporal parameters. The mean ± SD of SGPs of both groups are presented in Table 3.

## 4. Discussion

Our observational study aimed to compare postoperative SGPs in patients who received THA due to either primary hip OA or DDH and to explore any possible correlations with demographic or clinical variables. Our results showed that the values of SGPs of the OA group were slightly better than those of the DDH group without revealing a statistically significant difference. The distinct pathomechanics of OA or DDH associated with preoperative alterations in gait improved after THA to similar levels in both groups. As expressed by the OHS results, the OA group reported a better overall trend of the scores than the DDH group regarding self-estimated functionality. Significant correlations were found between the sample’s SGPs with age, the total OHS score, and four items of the OHS concerning hip pain and ADLs.

In the present study, the patients of the OA group were significantly older than the DDH group. This finding was expected since it is well-known that hip OA is a chronic disorder resulting from several distinct etiologic factors, including aging. Hip OA affects 7–25% of people older than 55 years [35] and demonstrates an increase in mean prevalence with advancing age [36]. On the other hand, DDH is the most common cause of secondary osteoarthritis in adults under 40 years of age, since abnormal hip biomechanics resulting in contact stresses predispose patients with DDH to arthritic changes earlier than the normal population and require THA at an early age [37]. Also expected was the fact that our groups consisted of more women than men, in line with studies reporting that hip OA prevalence is higher among women [38], while DDH is more common among girls [37]. Regarding age and sex, our groups were relatively representative of both populations studied [35,36,37,38]. 

The correlation analysis revealed statistically significant correlations between participants’ age, OHS scores (total and items), and the SGPs. Specifically, severe pain during long-time walking (item 5) was correlated with shorter stride length. Similarly, shorter step and stride length correlated with difficulty putting on socks, stockings, or tights (item 7). Additionally, the patient’s ability to perform household shopping independently (item 11) was correlated with faster walking speed, longer single support time, and shorter step time. On the other hand, a lower level of hip pain interference in usual work/housework (item 12) was linked to a slower cadence. Our findings support previous studies in which self-reported outcomes and biomechanical parameters were correlated in post-THA patients 12 months post-THA [21,22]. In the study of John et al. [21], the Hip Disability and Osteoarthritis Outcome Score (HOOS) correlated strongly with hip strength, while the correlations with step length asymmetry and contact time asymmetry were not significant and relatively weak (r < 0.32). In the study of Bolink et al. [22], moderate to strong significant correlations were found between the Western Ontario and McMaster Universities Osteoarthritis Index (WOMAC) and walking speed, cadence, and step time (0.31 < Pearson’s r < 0.51). Unfortunately, it is not possible to directly compare our OHS item results with the findings of the studies mentioned earlier, since the WOMAC and HOOS evaluate different aspects of pain and functionality than the OHS, which records experienced difficulty during a specific activity. However, correlations between self-reported outcomes and gait parameters may provide additional information showing how the latter affects post-THA-specific ADLs. These correlations can be used to develop personalized exercise programs for patients. By analyzing the data, healthcare professionals can identify specific areas of deficits and weaknesses in patients and create tailored exercise programs that target those areas. This approach helps reduce the deficits and improves the patient’s overall independence and quality of life.

No statistically significant differences between the two groups were observed in the SGPs. This can be explained by the effect of age as well as the lack of pre-operative data. However, our findings are consistent with the study conducted by Marangoz et al. [20], the only biomechanical study that directly compared the gait of post-THA OA and DDH patients [20]. Upon studying their results, we noticed that the average values of the SGPs of their groups were quite comparable to our findings. However, we did identify a difference in the walking speed and cadence of their DDH group, which were lower than the corresponding values we obtained in our study [20]. These differences might be due to the fact that their study’s gait analysis was carried out 12.5 months after THA, while in our study, the participants were measured after a three-and-a-half-year period. Studies have reported that after THA, the gait pattern generally improved significantly in all patients. However, patients with DDH tend to experience a more persistent pathological gait pattern, which subsides slowly over a more extended period [1,18]. This is due to the distorted hip anatomy (underdeveloped acetabulum and femur), LLD, decreased hip abduction range of motion, positive Trendelenburg sign, shortened iliopsoas, and hip adductors muscles that lead to asymmetrical gait than that of healthy controls. Patients tend to protect their DDH limb from childhood, and this compensation mechanism for the unaffected side in protecting the affected side remains after THA [19]. Therefore, it is suggested that a follow-up period longer than one year is necessary to obtain relevant results [39]. Extending the follow-up period beyond one year is essential to yield meaningful and insightful results. Thus, it is highly recommended that researchers extend their follow-up periods to achieve significant and relevant results [39].

Postoperative gait analysis is generally accepted as an objective measurement of surgical success since it effectively quantifies SGPs [20]. In addition to the objective gait assessment, the use of self-reported outcomes like OHS can provide unique information on the impact of treatment from the patient’s perspective [40], and it is complementary to the overall assessment of patients’ recovery; this is essential in clinical research and practice involving THA patients [40]. In our study, although the OA group had better outcome values (SGPs and OHS) than the DDH group, this was not reflected in the statistical analysis results, due to the lack of pre-THA data and the potential effect of age. However, these findings suggest that the pathological anatomy of DDH might be responsible for the observed phenomenon. Although the hip joint was reconstructed after THA, patients may continue to experience pain and discomfort on the affected side [19]. The possible reason is that in most DDH cases, widened intraoperative articular capsule release and tenotomies of the shortened hip muscles are advocated [28]. These necessary intraoperative soft tissue releases, combined with the aforementioned compensation mechanism of the unaffected side protecting the affected side, may impact the performance of daily activities in DDH patients, even after THA [19]. In order to minimize the soft tissue releases’ effects, studies suggest that patients with developmental dysplasia of the hip (DDH) can benefit from individualized exercise programs that prioritize strengthening the intact muscles in the lower limb. Specifically, exercises targeting hip flexors, hip abductors, and knee extensors have been effective [15,19]. 

To our knowledge, this is the second study that directly compares post-arthroplasty gait parameters between patients with primary OA and DDH patients. Our study supports the previous study’s findings [20], indicating that the distinct pathomechanics of OA or DDH, associated with pre-operative alterations in gait, improved after THA to similar levels in both groups. In addition, this is one of the very few studies [21,22] in which objective gait assessment via SGPs was correlated with patient-reported outcomes. These correlations can be utilized as a valuable tool for closely monitoring the progress of treatments within a clinical setting. Furthermore, they can significantly contribute to the advancement of tailored and personalized rehabilitation programs, ultimately enhancing the functional capacity and overall well-being of patients.

On the other hand, some limitations have to be mentioned. The main limitation is that this is a retrospective study of post-THA patients. Pre-operative data such as SGPs, LLD, Trendelenburg signs, possible muscle atrophies, or patient-reported outcomes were unavailable. Furthermore, the lack of pre-operative data regarding the correlations between age, SGPs, and OHS, as well as the lack of matching regarding age, prohibit us from conducting a more in-depth statistical analysis to explore group differences. It is important to note that the results of correlation analysis cannot be generalized due to the small sample size. Therefore, it is essential to interpret them with caution. Being mindful of this will lead to more accurate conclusions and better decision-making. More comparative and longitudinal biomechanical studies should be performed to improve the power of the current results and further investigate the postoperative gait of OA and DDH patients. 

Reflective surface markers are commonly used in traditional motion capture to assess joint kinematics. However, using skin markers on human tissue for motion analysis can introduce a possible source of measurement inaccuracy due to artifacts caused by the skin’s relative mobility compared to the underlying bone structures. Nonetheless, the literature strongly indicates that accurate and thorough tracking of gait analysis techniques minimizes any possible influence of errors on data collection when measuring kinetic and kinematic parameters with such equipment [41]. 

Future studies should be conducted while taking into account the potential effect of age when designing experimental protocols since, based on our results, age as a variable may influence the outcomes. Furthermore, combining kinematic and kinetic analysis with electromyography data studies can help evaluate the post-THA gait patterns of OA and DDH patients and optimize specific rehabilitation protocols. 

## 5. Conclusions

In our study, postoperative spatiotemporal parameter analysis after THA of OA patients and DDH patients revealed no significant statistical differences between groups, despite gait being slightly better in the OA group than the DDH group. Notably, there were significant correlations between post-arthroplasty SGPs and specific ADLs, suggesting that there may be a potential impact on the ability to perform specific activities. These findings should be correlated with kinetic gait analysis data to fully evaluate the differences in gait and functionality improvement after THA in these patient groups.

## Figures and Tables

**Table 1:** Demographic and clinical characteristics of the study’s sample (N = 30).

Characteristics	OA Group (N = 15)	DDH Group (N = 15)	*p*-Value
Age (years)	60.1 ± 3.82	46.13 ± 5.93	<0.005
Sex (Men/Women) [N (%)]	5(33.3%)/10(66.7%)	3(20%)/12(80%)	0.409
Height (cm)	163.95 ± 3.6	164.42 ± 3.1	0.181
Weight (kg)	69.35 ± 5.6	68.41 ± 4.5	0.135
Body Mass Index (kg/m^2^)	25.78 ± 2.6	25.30 ± 2.07	0.289
Years post-THA	3.91 ± 0.52	3.69 ± 0.52	0.123

The values for continuous variables are expressed as mean ± standard deviation (SD) and for categorical variables as frequencies (percentages).

**Table 2:** Item scores and overall Oxford Hip Scores (N = 30).

Items	OA Group (N = 15)	DDH Group (N = 15)
1. How would you describe the pain you usually have in your hip?	3.40 ± 0.74	3.33 ± 0.72
2. Have you had any trouble with washing and drying yourself (all over) because of your hip?	4.00 ± 0.00	3.93 ± 0.26
3. Have you had any trouble getting in and out of a car or using public transportation because of your hip? (whichever you tend to use)	3.66 ± 0.5	3.60 ± 0.49
4. Have you been able to put on a pair of socks, stockings or tights?	2.33 ± 0.47	2.47 ± 0.51
5. Could you do the household shopping on your own?	4.00 ± 0.00	3.86 ± 0.26
6. For how long have you been able to walk before the pain in your hip becomes severe? (with or without a walking aid)	3.60 ± 0.51	3.13 ± 0.77
7. Have you been able to climb a flight of stairs?	3.20 ± 0.40	3.07 ± 0.26
8. After a meal (sat at a table), how painful has it been for you to stand up from a chair because of your hip?	3.36 ± 0.49	3.53 ± 0.52
9. Have you been limping when walking, because of your hip?	3.13 ± 0.61	3.00 ± 0.55
10. Have you had any sudden, severe pain—“shooting”, “stabbing”, or “spasms”)—from your affected hip?	3.40 ± 0.52	3.20 ± 0.91
11. How much has pain from your hip interfered with your usual work (including housework)?	3.8 ± 0.47	3.53 ± 0.75
12. Have you been troubled by pain from your hip in bed at night?	3.47 ± 0.72	3.06 ± 0.72
Oxford Hip Score (total score)	41.67 ± 2.19	39.73 ± 1.58

The values are expressed as mean ± standard deviation (SD).

**Table 3:** Spatiotemporal parameters of the study’s sample (N = 30).

Parameters	OA Group (N= 15)	DDH Group (N= 15)
Walking speed (cm/s)	77.26 ± 4.83	74.75 ± 3.24
Cadence (steps/min)	94.69 ± 2.73	92.93 ± 3.17
Double support time (% cycle)	33.18 ± 2.13	31.78 ± 3.81
Single support (% cycle)	37.09 ± 3.8	35.12 ± 5.57
Step time (s)	0.66 ± 0.08	0.72 ± 0.11
Step length (cm)	48.62 ± 2.84	47.18 ± 2.87
Stride time (s)	1.23 ± 0.20	1.27 ± 0.11
Stride length (cm)	97.83 ± 5.45	95.45 ± 5.65

The values are expressed as mean ± standard deviation (SD).

## Data Availability

The data that support the findings of this study are available from the corresponding author, S.S., upon reasonable request. Data are available only on request due to privacy and ethical issues.

## Supplementary Materials

The following supporting information can be downloaded at: https://www.mdpi.com/article/10.3390/jfmk9030110/s1.
