# Supplementary material for: Post-Arthroplasty Spatiotemporal Gait Parameters in Patients with Hip Osteoarthritis or Developmental Dysplasia of the Hip: An Observational Study
Source: J Funct Morphol Kinesiol. 2024 Jun 25;9(3):110. doi: 10.3390/jfmk9030110 (PMC11270259; doi:10.3390/jfmk9030110)
Supplement: Supplementary file 1 [file jfmk-09-00110-s001.zip › jfmk-3062567-supplementary.pdf]

Post-arthroplasty spatiotemporal gait parameters in patients with hip osteoarthritis or developmental dysplasia of the hip: an observational study

	Item No.	Recommendation	Page No.	Relevant text from manuscript
**Title and abstract**	1	(*a*) Indicate the study's design with a commonly used term in the title or the abstract	1	Title: Post-arthroplasty spatiotemporal gait parameters in patients with hip osteoarthritis or developmental dysplasia of the hip: **an observational study**
		(*b*) Provide in the abstract an informative and balanced summary of what was done and what was found		This study compared post-THA spatiotemporal gait parameters (SGPs) between OA and DDH patients and explored correlations with demographic and clinical variables. Thirty patients (15 per group) were recorded during gait, and their SGPs were analyzed. Functionality was evaluated with the Oxford Hip Score (OHS). The OA patients were significantly older than DDH ($p<0.005$). There were no significant statistical differences in SGPs, although the OA group was marginally faster than the DDH group ($p\leq0.057$). Significantly moderate to strong were the correlations between SGPs, age, and four items of OHS concerning hip pain and activities of daily life ($0.31 >r< 0.51$, all $p< 0.05$). Following THA, both groups exhibited similar levels of the examined gait parameters. Post-arthroplasty SGPs and OHS correlations indicate limitations in certain activities.
**Introduction**				
Background/rationale	2	Explain the scientific background and rationale for the investigation being reported	2	However, the literature review revealed that in most gait analysis studies concerning the post-THA gait, either in patients with hip OA or DDH patients, the comparison was carried out with non-operated or with healthy peers [16, 19]. Up to our knowledge only one study compares post-arthroplasty gait parameters between patients with primary OA and DDH patients [20]. Therefore, the present study aims to compare the post-arthroplasty gait spatiotemporal parameters in patients with primary OA and patients with DDH.
Objectives	3	State specific objectives, including any prespecified hypotheses	2	The primary study hypothesis is that the distinct pathomechanics associated with each condition contribute to the preoperative adaptations in gait. Consequently, it is thought that there may be variations in the improvement of postoperative spatiotemporal characteristics. Secondary outcomes included potential correlations be-tween postoperative spatiotemporal and demographic/clinical characteristics relating to patients' functionality. Based on previous relevant studies on post-THA patients [21, 22], we hypothesize that postoperative spatiotemporal characteristics have the same trend with patient-reported outcomes.
**Methods**				
Study design	4	Present key elements of study design early in the paper	2	Trial design: The research was conducted in a biomechanics laboratory (Ethics Approval No: 42609/05-05-2022). Patients who agreed to participate in the study were given written informed consent according to the principles of the Declaration of Helsinki and its later amendments [23]. The present study conformed to the "Strengthening the Reporting of Observational Studies in Epidemiology" (STROBE) statement for reporting observational studies [24].
Setting	5	Describe the setting, locations, and relevant dates, including periods of recruitment, exposure, follow-up, and data collection	3	Information was obtained by reviewing the registry data from their admission for THA surgery and by conducting telephone interviews. After the first screening, the enrolled patients were divided into two groups according to their preoperative diagnosis. The first group (OA group) included patients who underwent THA due to unilateral hip OA, and the second group (DDH group) included patients who underwent THA due to secondary degenerative arthropathy due to unilateral DDH.
Participants	6	(*a*) *Cohort study*—Give the eligibility criteria, and the sources and methods of selection of participants. Describe methods of follow-up<br>*Case-control study*—Give the eligibility criteria, and the sources and methods of case ascertainment and control	2-3	Patients over 45 were included in the present study, as it has been reported that one in four arthroplasties performed before age fifty is due to hip dysplasia [25]. All participants underwent a cementless THA through a posterior approach [27, 28] performed by the same team of orthopaedic surgeons and all patients followed the same postoperative physiotherapy program. ……..Patients were excluded from the study if they had had previous hip joint preserving procedures or acquired

		selection. Give the rationale for the choice of cases and controls<br>*Cross-sectional study*—Give the eligibility criteria, and the sources and methods of selection of participants		post-THA a leg-length discrepancy (LLD) greater than 2 cm, a nerve injury, a history of other orthopedic surgery on the lower limbs or spine, declared that they suffer from a severe balance disorder, or neurological and musculoskeletal diseases that prevent them from performing free walking, or use a walking aid.
		(*b*) *Cohort study*—For matched studies, give matching criteria and number of exposed and unexposed<br>*Case-control study*—For matched studies, give matching criteria and the number of controls per case		
Variables	7	Clearly define all outcomes, exposures, predictors, potential confounders, and effect modifiers. Give diagnostic criteria, if applicable	3	Initially, the demographic characteristics (age, gender, height, weight, body mass index) of the two groups' populations were recorded. The preoperative grade of hip OA was recorded according to the Kellgren-Lawrence classification system [29], and the grade of DDH according to the Crowe classification system [30]. Anthropometric data were collected using a Seca scale (model 803) and a height meter. The knee and ankle joints' diameters, anterior superior iliac spine (ASIS) distance, and pelvic depth were measured with a caliper.<br>Patients' functionality was measured using the Oxford Hip Score (OHS), which consisted of 12 questions assessing pain and function during activities of daily living (ADLs). The OHS questionnaire was designed and developed to assess patients under-going THA [31]. Items' response scores range from 0 points (most severe symptoms) to 4 points (least symptoms), with a total score between 40 and 48 indicating satisfactory joint function [32].
Data sources/ measurement	8*	For each variable of interest, give sources of data and details of methods of assessment (measurement). Describe comparability of assessment methods if there is more than one group	3-4	2.4 Instrumentation and procedure<br>A motion recording system with six Vicon MCam optoelectronic cameras (Oxford MetricsGroup Ltd.) was used to record the patients' spatiotemporal parameters, which were recorded during walking.<br>The equipment was calibrated every morning by the same Biomechanist before the measurements, according to the applicable local protocols, to ensure accuracy and enable the calculation of each marker's three-dimensional (3D) coordinates. The mean error in calculating the difference between the measured and actual distance of two markers fixed to the ends of a rigid rod 600mm apart was within 0.3mm. The calibrated volume for this application was 10m in length (x-axis of the laboratory reference system), 3m in height (y-axis of the laboratory reference system), and 3m along the z-axis of the laboratory reference system. Records of these checks and associated calibrations were saved along with all session data.<br>All six optoelectronic cameras also used a frequency of 120Hz for data acquisition, while the motion analysis system error was <0.1mm in a 10 x 3 x 3m laboratory space volume (Figure 1). These calibration parameters also ensured the accuracy of the recorded data.<br>2.5 Modeling – placement of markers<br>Motion modeling is an essential concept in the field of biomechanical data record-ing. The Plug-in Gait marker fitting procedure was employed due to this rationale [33]. Markers were strategically positioned in the anatomical areas of the pelvis and lower extremities. The pelvic markers were placed at the anatomical landmarks of the left anterior superior iliac spine (LASI marker), the right anterior superior iliac spine (RASI marker), the left posterior superior iliac spine (LPSI marker), and the right posterior superior iliac spine (RPSI marker). As for the lower extremities, both the left and right, the following markers were positioned: on the upper lateral 1/3 area of the left and right thigh (LTHI/RTHI markers), on the flexion-extension axis of the left and right knee (LKNE/RKNE markers), on the lower 1/3 area of the left/right shank (LTIB/RTIB markers). To reconstruct the foot section, markers were positioned on the left/right lateral malleolus, passing along an imaginary line across the left/right transmalleolar axis (LANK/RANK markers), on the left/right calcaneus bone (LHEE/RHEE markers), and on the left/right second metatarsal head, on the mid-foot side of the equinus break between the fore-foot and mid-foot (LTOE/RTOE markers).<br>To achieve precise localization and positioning of knee markers (LKNE, RKNE), a slight passive

				flexion and extension of the knee were performed while carefully observ-ing the lateral knee joint skin area. The location where the knee joint's axis intersects the knee's outer surface was identified by locating the layer of skin on the thigh that moved the least. This landmark was designated with a pen as the focal point for the rotational movement of the foot's bottom.<br>Thigh markers (LTHI and RTHI) are utilized to identify the location of the knee flexion axis. The LTHI marker was positioned on the lower one-third of the outside lat-eral area of the thigh, while the RTHI marker was put on the upper one-third of the outer lateral surface of the thigh, slightly under the arm's reach point. However, the exact height of the markers is not an essential factor in this measurement. Proper identification of the knee flexion axis relies on the reflectors' anteroposterior location. The thigh marker's location was modified to align with the plane, including the hip and knee joints center and the axis representing knee flexion and extension.<br>The alignment of the plantar flexion axis is determined using tibial markers, namely the LTIB and RTIB. The LTIB marker was positioned on the lower one-third of the tibial surface, while the RTIB marker was placed on the upper one-third of the tibial surface, like the thigh markers. The tibial marker was positioned inside the plane, including the center of the knee and ankle joints and the axis representing ankle flexion and extension.<br>The participants conducted the walking process during a single laboratory session. They were instructed to walk in a manner that closely resembled their usual walking style, with occasional cues given, for a distance of approximately 6 meters at a self-chosen tempo. A preliminary static trial was conducted to establish the orientations of the markers before processing the model. Subsequently, participants performed two dynamic trials to familiarize themselves with the testing processes. Ultimately, they completed three additional trials that were considered sufficient and were then analyzed to obtain the representative values of the spatiotemporal parameters [19]<br>*2.6 Data synthesis*<br>Anthropometric measurements were combined with data from markers' deflections. All markers' location data were performed using Nexus 2.3 software. The spatiotemporal parameters measured in this study were walking speed, cadence, double support time, single support, step time and length, and stride time and length.<br>The above spatiotemporal parameters were included in the statistical analysis and were calculated using inverse dynamics and normalization in terms of body mass and length [34].
Bias	9	Describe any efforts to address potential sources of bias		The participants were enrolled in a non-randomized way and both patients and authors were not blinded, a fact that may predispose biased conclusions.
Study size	10	Explain how the study size was arrived at	5	A total of 50 patients were enrolled in the present study (the minimum required sample after Power Analysis was found to be 29 subjects in total).
Quantitative variables	11	Explain how quantitative variables were handled in the analyses. If applicable, describe which groupings were chosen and why	5	Pearson's r correlation index assessed correlations between continuous variables (demographic, clinical, and spatio-temporal parameters data) of all patients. Group differences assessed using ANVOVA.
Statistical methods	12	(*a*) Describe all statistical methods, including those used to control for confounding	4-5	Data were expressed for continuous variables as mean±standard deviation (SD) and for categorical variables as frequencies (percentages).<br>Normality was assessed by Q-Q plot inspection. Pearson's r correlation index assessed correlations between continuous variables (demographic, clinical, and spatio-temporal parameters data) of all patients. Group differences assessed using ANVOVA.<br>All tests were two-sided, with the significance level being p=0.05. All tests were performed using SPSS v.29 (IBM Corporation, Somers, NY, USA).

Continued on next page

		(*b*) Describe any methods used to examine subgroups and interactions	5	Given the absence of pre-THA data, the correlation between age and SGPS and OHS group differences were assessed using analysis of covariance (ANCOVA).<br>After controlling for age revealed (Pillai's trace =0.05, Wilk's Lamba =0.05) the ANCOVA showed that age adjusts the values of the outcomes (SPGs, OHS). Addition-ally, the multivariate test for the OA and DDH groups (Pillai's trace =0.651, Wilk's Lamba=0.651) indicates no significant main effect amongst the independent groups of the outcome mentioned above when controlling for age. The above indicates that age does indeed adjust the results, but there are no statistically significant main effects between the two groups, which is a significant finding.
		(*c*) Explain how missing data were addressed		
		(*d*) *Cohort study*—If applicable, explain how loss to follow-up was addressed<br>*Cross-sectional study*—If applicable, describe analytical methods taking account of sampling strategy	6	The Q-Q plot inspection revealed that variables had normal distribution; hence parametric testing was performed.
		(*e*) Describe any sensitivity analyses		NA
**Results**				
Participants	13*	(a) Report numbers of individuals at each stage of study—eg numbers potentially eligible, examined for eligibility, confirmed eligible, included in the study, completing follow-up, and analysed	5	A total of 50 patients were enrolled in the present study (the minimum required sample after Power Analysis was found to be 29 subjects in total). Of the 50 patients, 25 were diagnosed with hip OA before THA, and 25 had unilateral DDH. Ten did not meet the inclusion criteria, nine refused to participate, and one hip OA patient had passed away because of a cause unrelated to THA. Finally, 30 patients (15 in each group) were included. The detailed procedure of the participants' selection is presented in a flow diagram (Figure 2).
		(b) Give reasons for non-participation at each stage	5	Ten did not meet the inclusion criteria, nine refused to participate, and one hip OA patient had passed away because of a cause unrelated to THA
		(c) Consider use of a flow diagram	6	Flow diagram (Figure 2)
Descriptive data	14*	(a) Give characteristics of study participants (eg demographic, clinical, social) and information on exposures and potential confounders	5-6	The mean ±SD of the demographic and clinical characteristics of the study's sample were presented in Table 1. There were no significant demographic or clinical differences between the groups, except for age ($p<0.005$). When the THA was performed, the mean age of the OA group was 60.1 years (min=53, max=68), and the mean age of the DDH group was 46.13 years (min=36, max= 55 years). The OA group includes five men and ten women, while the DDH group consists of three men and 12 women. Eight patients of the OA group underwent THA due to grade III and seven due to grade IV hip OA, according to the Kellgren-Lawrence classification system. According to the Crowe classification system, the DDH group included four patients with Crowe II, six with Crowe III, and four with Crowe IV dysplastic hip. The greater preoperative LLD of DDH group was 5cm, while the hip OA preoperative LLD was not reported in the record files. The means of the post-THA time period were for the hip OA group 3.91 years (min=3.3, max=5) and for the DDH group 3.69 years (min=3.1, max=4.8).
		(b) Indicate number of participants with missing data for each variable of interest		None
		(c) *Cohort study*—Summarise follow-up time (eg, average and total amount)		
Outcome data	15*	*Cohort study*—Report numbers of outcome events or summary measures over time		
		Case-control study—Report numbers in each exposure category, or summary measures of exposure		
		Cross-sectional study—Report numbers of outcome events or summary measures	7-8	The OHS total score ranged from 38 to 42 in the OA group and 37 to 42 in the DDH group. The mean ± SD of the item scores and the total Oxford Hip Score of both groups are included in Table 2. No significant statistical differences were also observed between the two groups regarding their spatiotemporal parameters. The mean ±SD of SGPs of both groups are presented in Table 3.

Continued on next page

Main results	16	(*a*) Give unadjusted estimates and, if applicable, confounder-adjusted estimates and their precision (eg, 95% confidence interval). Make clear which confounders were adjusted for and why they were included		The age
		(*b*) Report category boundaries when continuous variables were categorized		
		(*c*) If relevant, consider translating estimates of relative risk into absolute risk for a meaningful time period		
Other analyses	17	Report other analyses done—eg analyses of subgroups and interactions, and sensitivity analyses	7-8	The correlation analysis revealed significant moderate to strong correlations. Specifically, moderate positive were the correlations between age and walking speed, step length, and total OHS score (r=0 p=0.00, r=0.34, p=0.00, and r=0.36, p=0.04. respectively), while negative and strong was the correl between age and step time (r= -0.51, p=0.04). Positive and moderate correlations were found betwee walking speed and item 5 of the OHS ("Could you do the household shopping on your own?") (r=0. p=0.02) and between cadence and item 11 of the OHS ("How much has pain from your hip interfere your usual work, including housework?") (r=0.38, p=0.03). The single support time positively and n correlated with item 5 of the OHS (r=0.46, p=0.01). Strong and negative was the correlation betwee time and item 5 of the OHS (r=-0.51, p=0.00). The step length was moderate and negatively correlat item 4 of the OHS ("Have you been able to put on a pair of socks, stockings or tights?") (r=-0.41, p= while the stride length was moderate and negatively correlated with item 6 ("For how long have you to walk before the pain in your hip becomes severe (with or without a walking aid)?") (r=-0.36, p=0 with item 4 (r=-0.44, p=0.01) of the OHS.
**Discussion**				
Key results	18	Summarise key results with reference to study objectives	8-9	The correlation analysis revealed statistically significant correlations between participants' age, OHS scores (total and items) and the SGPs. Specifically, severe pain during long-time walking (item 5) was correlated with shorter stride length. Similarly, shorter step and stride length correlated with difficulty putting on socks, stockings, or tights (item 7). Additionally, the patient's ability to perform household shopping independently (item 11) was correlated with faster walking speed, longer single support time, and shorter step time. On the other hand, a lower level of hip pain interference in usual work/housework (item 12) was linked to a slower cadence. Our findings support previous studies in which self-reported outcomes and biomechanical parameters were correlated in post-THA patients 12 months post-THA [21, 22]. In the study of John et al. [21], the Hip Disability and Osteoarthritis Outcome Score (HOOS) correlated strongly with hip strength, while the correlations with step length asymmetry and contact time asymmetry were not significant and relatively weak (r < 0.32). In the study of Bolink et al. [22], moderate to strong significant correlations were found between the Western Ontario and McMaster Universities Osteoarthritis Index (WOMAC) and walking speed, cadence, and step time (0.31 > r <0.51). Unfortunately, it is not possible to directly compare our OHS item results with the findings of the studies mentioned earlier, since WOMAC and HOOS evaluate different aspects of pain and functionality than OHS, which records experienced difficulty during a specific activity. However, correlations between self-reported outcomes and gait parameters may provide additional information showing how the latter affects post-THA-specific ADLs. These correlations can be used to develop personalized exercise programs for patients. By analyzing the data, healthcare professionals can identify specific areas of deficits and weaknesses in patients and create tailored exercise programs that target those areas. This approach helps reduce the deficits and improves the patient's overall independence and quality of life.<br>No statistically significant differences between the two groups were observed in the SGPs. This can be explained by the effect of age as well as the lack of pre-operative data. However, our findings are consistent with the study conducted by Marangoz et al. [20], the only biomechanical study that directly compared the

				gait of post-THA OA and DDH patients [20]. Upon studying their results, we noticed that the average values of the SGPs of their groups were quite comparable to our findings. However, we did identify a difference in the walking speed and cadence of their DDH group, which were lower than the corresponding values we obtained in our study [20]. These differences might be due to the fact that this study's gait analysis was carried out 12.5 months after THA, while in our study, the participants were measured after a three-and-a-half-year period. Studies have reported that after THA, the gait pattern generally improved significantly in all patients. However, patients with DDH tend to experience a more persistent pathological gait pattern, which subsides slowly over a more extended period [1, 18]. This is due to the distorted hip anatomy (underdeveloped acetabulum and femur), LLD, decreased hip abduction range of motion, positive Trendelenburg sign, shortened iliopsoas, and hip adductors muscles that lead to asymmetrical gait than that of healthy controls. Patients tend to protect their DDH limb from childhood, and this compensation mechanism for the unaffected side in protecting the affected side remains after THA [19]. Therefore, it is suggested that a follow-up period longer than one year is necessary to obtain relevant results [39]. Extending the follow-up period beyond one year is essential to yield meaningful and insightful results. Thus, it is highly recommended that researchers extend their follow-up periods to achieve significant and relevant results [39].<br>Postoperative gait analysis is generally accepted as an objective measurement of surgical success since it effectively quantifies SGPs [20]. In addition to the objective gait assessment, the use of self-reported outcomes like OHS can provide unique information on the impact of treatment from the patient's perspective [40], and it is complementary to the overall assessment of patients' recovery; this is essential in clinical research and practice involving THA patients [40]. In our study, although the OA group had better outcomes' values (SGPs and OHS) than DDH group this was not reflected in the statistical analysis results, due to the lack of pre-THA data and the potential effect of age. However, these findings suggest that the pathological anatomy of DDH might be responsible for the observed phenomenon. Although the hip joint was reconstructed after THA, patients may continue to experience pain and discomfort on the affected side [19]. The possible reason is that in most DDH cases, widened intraoperative articular capsule release and tenotomies of the shortened hip muscles are advocated [28]. These necessary intraoperative soft tissue releases, combined with the aforementioned compensation mechanism of the unaffected side protecting the affected side, may impact the performance of daily activities in DDH patients, even after THA [19]. In order to minimize the soft tissue releases' effects, studies suggest that patients with developmental dysplasia of the hip (DDH) can benefit from individualized exercise programs that prioritize strengthening the intact muscles in the lower limb. Specifically, exercises targeting hip flexors, hip abductors, and knee extensors have been effective [15, 19].
Limitations	19	Discuss limitations of the study, taking into account sources of potential bias or imprecision. Discuss both direction and magnitude of any potential bias	10	On the other hand, some limitations have to be mentioned. The main limitation is that this is a retrospective study of post-THA patients. Pre-operative data such as SGPs, LLD, Trendelenburg signs, possible muscle atrophies, or patient-reported outcomes were unavailable. Furthermore, the lack of pre-operatively data regarding the correlation between age, SGPs and OHS as well as the lack of matching regarding age, prohibit us from conducting a more in-depth statistical analysis to explore group differences. It is important to note that the results of correlation analysis cannot be generalized due to the small sample size. Therefore, it is essential to interpret them with caution. Being mindful of this will lead to more accurate conclusions and better decision-making. More comparative and longitudinal biomechanical studies should be performed to improve the power of the current results and further investigate the postoperative gait between OA and DDH patients. Reflective surface markers are commonly used in traditional motion capture to assess joint kinematics. However, using skin markers on human tissue for motion analysis can introduce a possible source of measurement inaccuracy due to artifacts caused by the skin's relative mobility compared to the underlying bone structures. Nonetheless, the literature strongly indicates that accurate and thorough tracking of gait analysis techniques minimizes any possible influence of errors on data collection when measuring kinetic and kinematic parameters with such equipment [41].

Continued on next page

Interpretation	20	Give a cautious overall interpretation of results considering objectives, limitations, multiplicity of analyses, results from similar studies, and other relevant evidence	10	It is important to note that the results of correlation analysis cannot be generalized due to the small sample size. Therefore, it is essential to interpret them with caution. Being mindful of this will lead to more accurate conclusions and better decision-making. More comparative and longitudinal biomechanical studies should be performed to improve the power of the current results and further investigate the postoperative gait between OA and DDH patients. The participants were enrolled in a non-randomized way and both patients and authors were not blinded, a fact that may predispose biased conclusions. It is important to note that the results of correlation analysis cannot be generalized due to the small sample size. Therefore, it is essential to interpret them with caution.
Generalisability	21	Discuss the generalisability (external validity) of the study results	10	The lack of pre-operatively data regarding the correlation between age, SGPs and OHS as well as the lack of matching regarding age, prohibit us from conducting a more in-depth statistical analysis to explore group differences. Future studies should be taking into account the potential effect of age when de-signing experimental protocols, since based on our results, age as a variable may influence the outcomes. Furthermore, combining kinematic and kinetic analysis with electromyography data studies can help evaluate the post-THA gait patterns of OA and DDH patients and optimize specific rehabilitation protocols.
**Other information**				
Funding	22	Give the source of funding and the role of the funders for the present study and, if applicable, for the original study on which the present article is based	10	This research received no external funding.

*Give information separately for cases and controls in case-control studies and, if applicable, for exposed and unexposed groups in cohort and cross-sectional studies.

Note: An Explanation and Elaboration article discusses each checklist item and gives methodological background and published examples of transparent reporting. The STROBE checklist is best used in conjunction with this article (freely available on the Web sites of PLoS Medicine at http://www.plosmedicine.org/, Annals of Internal Medicine at http://www.annals.org/, and Epidemiology at http://www.epidem.com/). Information on the STROBE Initiative is available at www.strobe-statement.org.
